# The Present Hospital at Melbourne

**Published:** 1911-05-27

**Authors:** 


### INSTITUTIONAL LETTER-BOX.

### THE PRESENT HOSPITAL AT MELBOURNE.

*To the Editor of* The Hospital.

Dear Sir,—You may be interested to learn that Mr. Westmore Stephens, a governor of the Melbourne Hospital, is about to pay a visit to Europe, during which he will devote some time to the study of various hospital arrangements. It is to be hoped that our hospital administrators will welcome and give him such facilities as will aid him in his intention, which is, of course, to benefit the Melbourne Hospital, an institution in a pitiable condition when I visited it in 1906. Since then the matron has been in this country, and doubtless has instituted improvements. At the date of my visit the condition of the hospital as a whole was a reproach to any large city, and especially to a prosperous modern city such as Melbourne. In fact it would have been difficult to find its equal in any English-speaking country. In one ward I visited there were large holes in the time-worn and unsightly flooring-boards. These, it was explained to me, were the work of rats. The crockery of the same ward consisted of enamelled ware, on which little enamel remained, and which, apart from the appearance of squalor, was highly objectionable on sanitary grounds. One or two wards I saw were a decided improvement on the first, but none came up to the standard regarded as essential in a modern hospital, and the general impression that I carried away was that there was no hope of attaining real efficiency in the hospital, but that new buildings and new site were alike imperative. Although both Secretary and Matron were on the premises at the time of my visit, the office of cicerone was relegated to a probationer, and I may therefore have missed seeing some possible feature of special interest in the buildings. But even if such exist, the value must be so discounted that they shoul dbe sacrificed without hesitation. Melbourne should not be content until it possesses a hospital as good as the one at Sydney. There the older institution—the Sydney General Hospital—is superior in every respect, greatly superior; and Sydney possess in the Royal Prince Alfred Hospital an institution which might serve as a model anywhere—excellently situated, splendidly planned, equipped, and administered—a hospital any city should be proud to own, the like of which Melbourne should strive to attain.

Mr. W. Epps, the able and energetic Secretary, received every assistance when he visited this country on a tour of hospital inspection, and I feel sure that Mr. Westmore Stephens will be welcomed with equal courtesy and cordiality.—Yours faithfully, A Subscriber.

### HOSPITALS AND FLANNELETTE.

*To the Editor of* The Hospital.

